# Extracellular Vesicles as Innovative Treatment Strategy for Amyotrophic Lateral Sclerosis

**DOI:** 10.3389/fcell.2021.754630

**Published:** 2021-11-11

**Authors:** Ke Wang, Yu Li, Chao Ren, Yongjing Wang, Wenshan He, Yuan Jiang

**Affiliations:** Clinical Medical College and The First Affiliated Hospital of Chengdu Medical College, Chengdu, China

**Keywords:** amyotrophic lateral sclerosis, extracellular vesicles, treatment strategy, exosome-mimics, plant EVs

## Abstract

Amyotrophic lateral sclerosis (ALS) is a progressive and fatal motor neuron degenerative disease, and it is hard to diagnose in the early stage, and treatment means are limited, and the treatment effect is unsatisfactory. Therefore, exploring a new effective treatment strategy is urgently needed for ALS patients. Extracellular vesicles (EVs) are a heterogeneous group of natural membrane vesicles containing many bioactive substances, and they play important roles in the paracrine pathway and exhibit neuroprotection effects. A growing body of evidence shows that EVs have great application potential in diagnosis, treatment, and drug delivery in ALS, and they represent an innovative treatment strategy for ALS. In this review, we will briefly introduce the biogenesis of EVs and focus on discussing the role of EVs in ALS treatment to further enrich and boost the development of EVs as an innovative treatment strategy for ALS.

## Introduction

Amyotrophic lateral sclerosis (ALS), also known as Lou Gehrig’s disease, is a fatal neurodegenerative disease that results from selective dysfunction and damage of upper and lower motor neurons (MNs) and leads to progressive paralysis and death. An estimated that the worldwide prevalence of ALS is 5 cases per 100,000 population, and the incidence is approximately 2 per 100,000 individuals per year (Christoforidou et al., 2020). In 90–95% of ALS patients are sporadic (sALS) who have no apparent genetic link, and 5–10% are familial (fALS) who have a family history (Bonafede and Mariotti, 2017; Ferrara et al., 2018). The first symptoms of fALS patients appear at the mean age of 50 and 60 years for sALS, and the characteristics of both sALS and fALS are selective degeneration of both upper motoneurons in the primary motor cortex and lower motoneurons in the brainstem and spinal cord, so the disease appears clinically indistinguishable (Bonafede and Mariotti, 2017). The clinical characteristic of ALS show that the disease begins focally (e.g., the distal end of the affected limb) and spreads during progression that will affect the muscles of mobility, speech, swallowing, and respiration, indicating motor neuron death from one starting motor unit to adjacent ones through a mechanism involving altered intercellular communication between neurons and glial cells (Garden and La Spada, 2012; Brettschneider et al., 2015). However, the pathogenetic mechanisms of ALS are still unclear (Paschon et al., 2016; Chen et al., 2019). Several altered signaling pathways were involved in the pathogenesis of ALS. For example, mitochondrial dysfunction, glutamate excitotoxicity, oxidative stress, protein aggregates, neurofilaments accumulation, and neuroinflammation (Bonafede and Mariotti, 2017; Calabria et al., 2019). Moreover, epigenetics, metabolic diseases, autoimmune pathology, lifestyle, and environmental factors, especially the exposure of heavy metals, solvents, pesticides, and chemicals, have been implicated in ALS (Maguire, 2017; Bendotti et al., 2020). Given the complexity of ALS pathogenesis, unfortunately, there are no effective treatment strategies that can bring anticipant benefits for disease course and survival of ALS patients (Kamelgarn et al., 2016). In general, the disease is fatal within 2–5 years after clinical onset, and about 50% of patients die within 30 months from symptom onset, while only less than 20% of patients may survive for more than 5 years (Grad et al., 2014a,b). Riluzole and edaravone are some of the few pharmacological agents for ALS, but they transiently attenuate disease progression for several months, and the therapeutic efficacy of ALS patients obtained in clinical trials is still controversial (Staff et al., 2019; Swindell et al., 2019). In the last years, gene therapy and stem cell therapy as the promising therapeutic approaches to get the attention of scholars. In particular, stem cell therapy can address cellular replacement and neural protection in different neurodegenerative diseases, including ALS (Staff et al., 2019). Previous studies demonstrated that mesenchymal stem cells (MSC) delay the death of motoneurons, decrease the inflammatory response and prolong the survival time of the animal models after injections of MSC (Wang et al., 2020). Even in cell-based clinical trials, MSC possesses the feasibility, safety, and immunological effects (Vinaiphat and Sze, 2019). However, many issues need to be resolved before extensive clinical translation, such as standardized protocols, including the route of administration, the dose of cells, the timing, and the number of cell injections (Staff et al., 2019). In recent years, some evidence indicates that only a small part of injected stem cells reach the lesion site, and proliferate and differentiate into effector cells. In the clinic, the practitioner usually has no way to determine the feasibility and therapeutic value of stem cell therapy (Maguire, 2017). Moreover, stem cells cannot cross the blood-brain barrier (BBB), so some scholars suggested that the beneficial effect of stem cells in neurodegenerative diseases may rely mainly on their paracrine activity rather than their engraftment (Bonafede et al., 2016), especially those cells that can produce extracellular vesicles (EVs) (Jin et al., 2021). Evidence shows that EVs play important roles in the paracrine pathway and exhibit neuroprotection effects (Lee and Kim, 2017).

Extracellular vesicles are a heterogeneous group of natural membrane vesicles containing many bioactive substances (e.g., proteins, lipids, and nucleic acids) released from various cells, including neurons, microglia, and astrocytes (Chen et al., 2019). EVs can be isolated from biological fluids, including cerebrospinal fluid (CSF), plasma, serum, breast milk, lymph, bile, and saliva (Izadpanah et al., 2018; Banack et al., 2020). According to their origin and size, EVs can be classified into three main subtypes, namely exosomes, microvesicles, and apoptotic bodies (Ferrara et al., 2018; Chen et al., 2019). EVs contain bioactive substances and transfer them between cells, resulting in those vesicles play the autocrine/paracrine role in intercellular communication. EVs involve various physiological and pathological processes (Jiang et al., 2020a). For example, EVs possess some function in the pathogenesis, diagnosis, and treatment strategy of some brain diseases, including ischemic stroke, Alzheimer’s disease, Parkinson’s disease, ALS, multiple sclerosis, and brain cancers. Thus EVs have received much attention (Silverman et al., 2016; Ciregia et al., 2017; Vinaiphat and Sze, 2019). Cu/Zn- superoxide dismutase one (SOD1) is one of the ALS-associated proteins, and mutant SOD1 can be released from ALS astrocytes through EVs (Gomes et al., 2007; Basso et al., 2013). Other ALS-associated proteins including TDP-43, FUS, neurofilament light chain (NfL) and INHAT repressor (NIR), also exist in the EVs, which are isolated from cerebrospinal fluid (CSF) or plasma of ALS patients (Sproviero et al., 2018; Chen et al., 2020; Hayashi et al., 2020; Vassileff et al., 2020b). In addition, some ALS-associated miRNAs and mRNAs also exist in the EVs of patients with ALS (Xu et al., 2018; Katsu et al., 2019; Otake et al., 2019; Saucier et al., 2019; Varcianna et al., 2019). In particular, exosomal inflammatory-related miRNAs induce a persistent NF-kB activation in microglial cells, which may results in aggravated microglia neurotoxicity toward MNs, and neuroinflammation in ALS patients (Pinto et al., 2017). Meanwhile, the change of proinflammatory mediators and cytokines can be shown by the exosomes from brain cells. Some scholars suggested that inflammatory biomarkers with increased levels in astrocyte-derived exosomes (ADEs) of sALS patients were positively associated with the rate of disease progression, for instance, interleukin-6 (IL-6) (Chen et al., 2019). In addition to exosomes, the microvesicles derived from leukocytes are mostly present in ALS patients. They also can serve as biomarkers to determine the stage of the disease due to they selectively transported the misfolded SOD1 (Sproviero et al., 2019). Therefore, EVs-mediated transfer of pathological proteins and genes are involved in the mechanism of ALS, and EVs are potentially a biomarker in the clinical diagnosis of ALS (Roy et al., 2019; Hayashi et al., 2020; Thompson et al., 2020).

Because of EVs serve as the carriers of pathological proteins and genes in ALS, some scholars suggested that different stages of exosome secretion pathways (e.g., vesicle formation, release, trafficking, and uptake) may be a therapeutic target. Inhibition of exosome secretion by pharmacological methods may provide some beneficial effects in neurodegenerative diseases (Dinkins et al., 2014; Asai et al., 2015; Gagliardi et al., 2021). However, Iguchi et al. (2016) suggested that this treatment strategy needs to be used with caution in ALS. EVs play key roles in nerve regeneration, neuronal protection, synaptic plasticity, and remyelination, thus EVs can be one of the new therapeutic options for neurodegenerative disease, especially EVs are regarded as the optimal carriers for functional RNAs (e.g., mRNAs and miRNA) in the transportation of genetic information between cells (Jin et al., 2021). Xin et al. (2012) first demonstrated that MSCs regulate neurite outgrowth and neural plasticity by transferring miR-133b to neural cells via the exosomes released from MSCs. In addition, other miRNAs, which are involved in cell proliferation and differentiation, were observed in the bone marrow-MSCs (BM-MSCs)-derived microvesicles and transferred to target cells, indicating that the biological effect of stem cells may, at least in part, depend on the EVs containing functional RNAs, and EVs may play a major role in the autocrine and paracrine regulation of development, differentiation, and cell survival (Collino et al., 2010). Thus, more and more scholars have realized the function of EVs and then shifted the focus from stem cells to their EVs to explore a non-cell-based therapy for ALS treatment (Bonafede and Mariotti, 2017).

In this review, we will briefly introduce the biogenesis of EVs and focus on discussing the role of EVs in ALS treatment to further enrich and boost the development of EVS as an innovative treatment strategy for ALS.

## Biogenesis of Extracellular Vesicles

**FIGURE 1:**
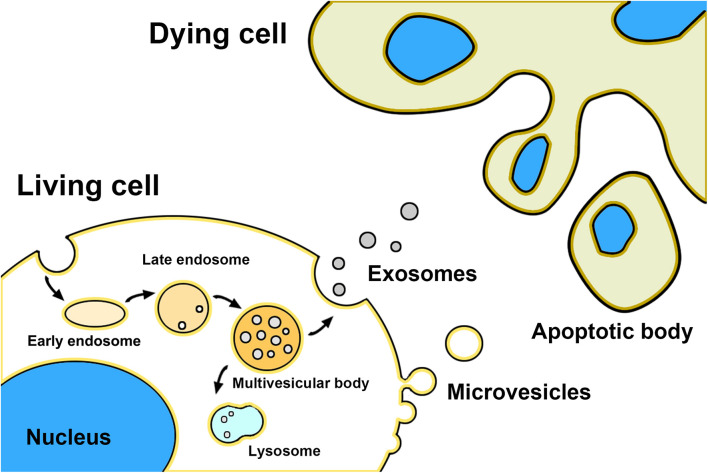
Schematic representation of EVs biogenesis.

Exosomes, microvesicles, and apoptotic bodies are the three main classes of EVs (Gharbi et al., 2020; Figure 1). Exosomes are nano-sized natural membrane vesicles with a diameter ranging from 30 to 100 nm and around double-membrane structures in appearance (Jiang et al., 2020b). Unlike other EVs, exosomes are formed from the internal membrane as early endosomes through the invagination and endocytosis of the plasma membrane and then matures into late endosomes. After encapsulating bioactive content (e.g., DNA, mRNA, miRNA, and proteins), late endosomes are budding into intraluminal vesicles (ILVs) and encapsulated into multivesicular bodies (MVBs) through the endosomal sorting complex required for transport (ESCRT)- dependent pathway or ESCRT -independent pathway (Coleman and Hill, 2015; Vassileff et al., 2020a). At last, MVBs fused with lysosomes resulting in the self-degradation, or with the plasma membrane resulting in the release of themself into the extracellular environment as exosomes (Andjus et al., 2020; Kutchy et al., 2020). Exosomes can be internalized into neighboring or distant cells by endocytosis, phagocytosis, and direct fusion with the plasma membrane, resulting in the alteration of the physiological state of recipient cells (Li et al., 2020). Microvesicles with a bigger size range (50–1000 nm) direct budding from the plasma membrane (Silverman et al., 2019). Biogenesis of microvesicles needs ARF6 and RhoA-dependent rearrangement of the actin cytoskeleton and the Ca2+ influx dependent activation of enzymes (Jiang et al., 2020a). Those enzymes disassemble the cytoskeleton at the formation site of microvesicles and change the lipid composition of the membrane, resulting in the reshaping and outward budding of the plasma membrane for microvesicles detachment. Moreover, some members of the ESCRT complex may also be involved in the microvesicles biogenesis, such as ESCRT-III (Jiang et al., 2020a). Other factors like cellular stress and immune response may also trigger microvesicles release (Gagliardi et al., 2021). Some studies indicated that microvesicles also transfer mRNA and miRNAs to recipient cells as well as exosomes, but the mechanisms of the sorting of RNAs in microvesicles biogenesis still need further to be explored. In addition, it is difficult to distinguish between exosomes and microvesicles by existing experimental methods because they have some overlaps in the biophysical characteristics and lack discriminating markers (Mathivanan and Simpson, 2009). Apoptotic bodies are the biggest EVs which sizes ranging from 500 to 5000 nm (Stolzing and Grune, 2004). Different from other EVs, apoptotic bodies are released from the cells undergoing programmed cell death and bearing nuclear fragments, chromatin, DNA, glycosylated proteins, and even organelle fragments to transfer their cargo to recipient cells, but the potential role in cell-cell communication is less studied (Bonafede et al., 2016; Andjus et al., 2020).

## Extracellular Vesicles as Therapeutic Agent for Amyotrophic Lateral Sclerosis

Exosomes are the smallest one of EVs and able to cross the BBB, indicating that exosomes can be a potential cell-free therapeutic agent in neurodegenerative diseases, such as ALS (Ciregia et al., 2017). EVs can be obtained from different cell types, especially MSCs can secrete a higher amount of EVs than other cell types, and those vesicles that shown promising effects in multiple conditions through triggering regeneration responses (Wang et al., 2020). Many research focuses on the possible therapeutic application of EVs derived from stem cells to ALS (Table 1), especially the exosomes derived from adipose-derived stem cells (ADSCs). Bonafede et al. (2016) intended to explore new options for ALS treatment to overcome obstacles and risks associated with the use of native or engineered stem cells. The motoneuron-like cell line NSC-34 was transfected with different SOD1 point mutations to mimic the behavior of ALS motoneurons and exposed to H_2_O_2_ in their study. They demonstrated for the first time that exosomes derived from ADSCs exert a neuroprotective role on NSC-34 cells overexpressing ALS mutations. Those exosomes can protect NSC-34 cells from oxidative damage and increasing cell viability, indicating the possible therapeutic application of ADSCs -derived exosomes for ALS. Bonafede and colleagues suggest that the beneficial effect of ASCs exosomes in ALS could be due to the regulatory role of exosomal miRNAs (e.g., miRNA21, miRNA222, and miRNAlet7a) in apoptosis-inhibiting pathway, cell cycle progression and proliferation (Bonafede et al., 2016). Lee et al. (2016) observed that ADSCs -derived exosomes reduce increased SOD-1 aggregation and modulate cellular phenotypes in the G93A ALS *in vitro* model, and some beneficial effects of ADSC-exosomes had embodied in the restoration of mitochondrial functions. Then, they suggested that ADSCs -derived exosomes can be a potential source of ALS treatment strategy, but its effects and safety need to be further confirmed before applications (Lee et al., 2016). The specific action of ASCs-exosomes on mitochondrial respiratory pathways remains to be clarified, Bonafede and colleagues further demonstrated that the expression of the mutated protein SOD1(G93A) in NSC-34 cells induced mitochondrial dysfunction, which has played a key role in the selective vulnerability of motoneurons in ALS in the subsequent research. They found that ADSCs-derived exosomes were able to revert the mitochondrial dysfunction, possibly because of the presence of the SOD1 in exosomes could counteract the mutated SOD1 protein. Those results provided additional evidence that EVs may be a viable treatment approach for ALS (Calabria et al., 2019). In addition, Bonafede et al. (2016) identified a total of 189 exosomal proteins were observed in ADSCs-derived exosomes using proteomic analysis, and those proteins were mainly involved in cellular pathways of cell adhesion and negative regulation of the apoptotic process. The protein network and pathway analysis showed that some major proteins were involved in the response of stress and the PI3K-Akt signaling pathway. Those results could elucidate the possible mechanism of neuroprotective effect and modulating the apoptotic pathway of ADSCs-derived exosomes *in vitro* ALS model (Bonafede et al., 2019). In the ALS mice model, Bonafede and colleagues observed that ADSCs-derived exosomes were able to reach the CNS and accumulated in the typical lesioned brainstem motor nuclei after intranasal (i.n.) administration. Repeated administration of ADSCs-derived exosomes was able to improve the motor performance and slow down the clinical progression of ALS rely on the protection of lumbar motoneurons, neuromuscular junction, and muscle, and inhibition of the activation of the glial cell in the treated ALS mice model. Moreover, Bonafede and colleagues suggested that administer ASC-exosomes every 4 days presents an optimal compromise between frequency and route of administration, which may avoid the endothelial injury of the ALS mice model by more frequent injections. These data showed that ASC-exosomes home to lesioned regions of the animal brain, and provide some evidence in the animal model for the promising use of ASC-exosomes in ALS. Of course, the mechanisms of ASC-exosomes uptake by brain cells need to be further explored that would contribute to open the way to the application of exosomes therapy in ALS (Bonafede et al., 2020). Garbuzova-Davis and Borlongan (2021) observed that the human bone marrow-derived endothelial progenitor cell (hBMEPC) -derived EVs can reduce the damage of endothelial cells (ECs) induced by ALS mouse plasma. Previous research demonstrated that the capillary alterations within the central nervous system (CNS) exist in ALS animal models and ALS patients. So the damaged capillary endothelium is inadequate for maintaining vascular homeostasis within and outside of the CNS, potentially representing an additional pathogenic mechanism of ALS. Hence, based on their findings, Garbuzova-Davis and Borlongan suggested that repair the altered endothelium by EVs derived from human stromal cells, and 1 μg/mL of EVs was beneficial in alleviating mouse brain endothelial cells (mBEC) damage by ALS mouse plasma. The above evidence has shown that the neurotherapeutic potentiality and successful application of EVs secreted by stem cells in ALS, indicating that EVs could be an safer non-cell therapy than stem cell therapy because it can have similar effects of stem cells without invasive methods and side effects of cell injection (Lee et al., 2016). EVs from stem cells represent a promising approach to treat ALS, but some areas still need elucidation, such as the EVs molecular content, specific EVs component, mechanisms of EVs uptake, and therapeutic effects of EVs administration into SOD1 mutant mice, that are involved in interfering with ALS pathogenesis (Wang et al., 2020; Garbuzova-Davis and Borlongan, 2021).

**TABLE 1:** Literature Examples of EVs derived from stem cells to treat ALS.

Donor cells	Type of model	Mode of action	References
Adipose-derived stem cells	Cell	Reduce oxidative damage	Bonafede et al., 2016
Adipose-derived stem cells	Cell	Restoration of mitochondrial functions	Lee et al., 2016
Adipose-derived stem cells	Cell	Restoration of mitochondrial functions	Calabria et al., 2019
Adipose-derived stem cells	Mice	Improve the motor performance	Bonafede et al., 2020
Human bone marrow-derived endothelial progenitor cell	Cell	Repair the altered endothelium	Garbuzova-Davis and Borlongan, 2021

## Extracellular Vesicles as Drug Delivery Vehicle for Amyotrophic Lateral Sclerosis

Brain drug delivery is one of the bottlenecks for the treatment of brain diseases (Zheng et al., 2019). Over 98% of small molecule drugs and almost 100% proteins, peptides and genes cannot penetrate the BBB. Hence, there is an urgent need to develop new therapeutic modalities to overcome BBB and improve efficacy (Niu et al., 2019). EVs are a kind of natural vesicles with double membrane structure, leading to EVs not only can carry biologically active molecules, but also keep these molecules from degrading in the extracellular environment, and facilitate the recipient cells uptake to achieve transfer of biological information over short and long distances to the recipient cells (Gomes et al., 2020; Wang and Zhang, 2020). EVs have cell-surface molecules and a high affinity for tissues, reducing the risk of off-target effects (Gagliardi et al., 2021). In addition to exchanging their naturally carried cargoes, exosomes can also deliver exogenous biomolecules into the target cells. EVs are gaining increasing attention as an ideal drug delivery system in neuronal disease treatment, especially their ability to cross the BBB (Yang et al., 2015; Chen et al., 2016; Matsumoto et al., 2017; Gassama and Favereaux, 2021). EVs can be manipulated and engineered to deliver exogenous molecule therapeutics, including small chemicals, nucleic acids, and proteins by chemical, biological, or physical methods (Bonafede et al., 2016). The engineered exosomes may greatly enhance therapeutic efficacy by maintaining molecule therapeutics *in vivo* integrity and improving biodistribution. Until now, the strategies of drug loading can be roughly divided into donor cells engineering and exosomes engineering. The donor cells engineering requires hijacking the endogenous loading machinery of the donor cells. Donor cells first absorb drug molecules, then produce and release exosomes containing drug molecules. By comparison, exosomes engineering is a more common strategy because the process is simple and controllable, and has relatively higher drug loading (Zheng et al., 2019). Co-incubation is the simplest method of exosomes engineering by incubating the isolated exosomes with drug molecules. For instance, the hydrophobic drugs (e.g., curcumin) can be loaded into exosomes after incubating for a few minutes, and then transported to the brain via a local intranasal administration (Sun et al., 2010; Zhuang et al., 2011). In addition, some active loading strategies, such as electroporation, sonication, freeze-thaw cycles, extrusion, and saponin, have been extensively applied to achieve a higher loading efficiency of hydrophilic drugs (Lunavat et al., 2016). The loading efficiency of the above methods is higher than co-incubation and can maintain the integrity of exosomes (Haney et al., 2015). Besides drug molecules, exogenous genetic material (e.g., miRNAs, mRNAs, and other small RNA) can also be loaded into exosomes. Small interfering RNA (siRNA) is the conventional treatment method of gene therapy by interfering with neurodegenerative disease-associated gene expression, but siRNA can hardly cross the BBB due to its bigger size. At present, more and more evidence support that EVs are the ideal carriers for siRNA-based gene therapy, and the EVs-based siRNA delivery system has strong therapeutic potential in neurodegenerative diseases. Electroporation is the most commonly used for siRNA incorporation into exosomes. This method can create many transient tiny pores on the exosome membrane under electrical current. Alvarez-Erviti et al. (2011) were the first to use electroporation to load exogenous siRNA into exosomes, and exosomes containing siRNA possess high delivery efficiency in neuronal cells (Neuro2A). Moreover, Didiot et al. (2016) demonstrated that hydrophobically modified siRNAs (hsiRNAs) had been efficiently loaded into exosomes after simply co-incubation, and exosomes as carriers increased hsiRNA stability and promoted the bilateral distribution in both striatal and cortical regions. Although the research of the exosomal siRNA delivery system is still in the early stage, existing research may take a solid step in the clinical application of siRNA (Niu et al., 2019). In recent years, scholars began to study the surface modification of exosomes by conjugated with targeting ligands (e.g., aptamers, antibodies, and peptides) through physical or chemical methods to improve exosomes’ intracellular uptake and ability to brain cells (e.g., neurons and astrocytes), and even cancer cells (Jia et al., 2018; Tian et al., 2018). Accumulating evidence suggests that EVs, either unmodified or engineered, can cross or bypass the BBB both *in vivo* and *in vitro*, meanwhile get access to the brain by crossing the cerebrospinal fluid-brain barrier in the choroid plexus (Grapp et al., 2013). Thus EVs as drug delivery vehicles for neurodegenerative disease therapies may be advantageous over classical synthetic drug delivery systems (Vinaiphat and Sze, 2019; Zheng et al., 2019). The synthetic nanocarriers, such as liposome vesicles and pegylated nanoparticles, may produce antibodies and induce short blood circulation half-lives after multiple dosing. Moreover, the toxicity and elimination process of the synthetic nanocarriers in the CNS has not been fully elucidated (Niu et al., 2019; Zheng et al., 2019). Recent advances in gene therapy (e.g., antisense oligonucleotides, and siRNA) renew hope for developing an effective method to treat ALS, but so far, there is no good effect in clinical trials. In particular, the BBB poses a significant challenge for effective gene therapy of ALS. Some scholars suggested that EVs can act as a non-viral vector for the delivery of genetic material to treat ALS due to their ability to transport various cellular entities across the BBB. Surprisingly, the research on using EVs for gene delivery in the treatment of ALS is very few (Ediriweera et al., 2021). Nevertheless, this aspect of research work has become an interesting subject with the thriving advances in nanotechnology.

## Exosome-Mimics and Plant Extracellular Vesicles

At present, the number of native exosomes produced by mammalian cells is limited, result in that large-scale production is a bottleneck problem in the clinical application of exosomes (Kalimuthu et al., 2018; Li et al., 2018). Cultured MSCs are often chosen as the donor cell in many research because they are the efficient producers of exosomes. However, one million MSC only produce less than 4 μg of exosomes per day (Kim et al., 2020). Exosome-mimics (EMs) are generated by cell extrusion or polymer nanoparticles coated with cell membranes (Li et al., 2018). The preferred method to generate EMs *in vitro* is mini-extruder-based technology, which is often used to produce liposomes loaded with therapeutics (Dong, 2018). In this method, the cells or plasma membrane are extruded by a lipid syringe extruder with serial pores size from 400 to 100 nm to generate spherical nanovesicles or membrane-enclosed polymer nanoparticles (Hu et al., 2011). The production of EMs is 100 times more than native exosomes, so those artificial vesicles are advantageous over native exosomes in clinical-scale production (Jang and Gho, 2014). Most notably, the EMs are not involved in any physiological mechanism of exosome biogenesis, especially cargo loading is not selected by specific mechanism unlike native exosomes, such as the ESCRT-dependent and lipid raft-dependent pathways, but they are similar to native exosomes in the vesicle structure and size, zeta-potential, biophysical characteristic, and therapeutic potential (Jang et al., 2013). Moreover, the EMs possessed the immunocompatibility and stability of native exosomes due to coating the plasma membrane, and can also be engineered through membrane modification to improve targeting ability and cellular uptake. Yang and colleagues are the first to load siRNA into EMs by electroporation. More than 15% of the siRNA was loaded into EMs, indicating that the gene transfer efficiency of EMs is good as well as native exosomes. Three endocytic pathways were involved in the EM uptake process in cancer cells, especially clathrin and caveolae-mediated endocytosis play the leading roles (Yang et al., 2016). Thus, the EMs are promising candidate delivery vehicles in ALS treatment.

The immortal cell lines (e.g., stem cells and cancer cells) are chosen as the donor cell to produce EVs in many research, but the EVs derived from those cells may carry carcinogenic substances to transmit pro-cancerous traits into recipient cells, indicating the biosafety concerns of EVs need to be concerned (Schillaci et al., 2017). In recent years, increasing evidence demonstrates that plant cells can produce EVs to regulate various biological functions (Cui et al., 2019). The EVs derived from plants have better safety profiles because plants do not harbor zoonotic or human pathogens (Dad et al., 2021). In contrast to mammalian cells, plant cells come from reliable and economically practical sources (Luan et al., 2017; Fernandes et al., 2020). Surface modification of plant EVs can also broaden the scope of desired target ability, so plant cells are considered as an alternative option for donor cells to produce EVs (Yepes-Molina et al., 2020). The exosomes derived from some plants (e.g., grapefruit, ginger, aloe, and Citrus limon L.) have been engineered to serve as drug delivery nanoplatforms for anti-tumors therapy (Dad et al., 2021). As one of them, grapefruit-derived EVs can carry exogenous miRNA (miR-17) and cross the BBB to achieve quick intracerebral tumor internalization for miR-17 delivery, result in downregulating the MHC class I expression of mouse brain tumor cells by the activation of natural killer cells, and inhibiting the tumor growth after intranasal administration (Zhuang et al., 2016). By comparison with artificially synthesized nanoparticles (e.g., liposomes and micelles), plant EVs also possess a lipid bilayer structure and the ability to deliver both hydrophilic and hydrophobic cargo. But they have low immunogenic effects, enhanced cellular uptake, higher stability in the gastrointestinal tract, and specific target ability (Fujita et al., 2018). In addition, the preparation method of a plant EV-based delivery system is less complex and faster than artificially synthesized nanoparticles (Fan and Zhang, 2013). Of course, our current knowledge of the physiology, cellular and molecular mechanisms of plant EVs is still limited. But it is undeniable that plant EVs have the profound potential of implementation into the drug delivery system, and those vesicles might provide more opportunities for ALS treatment.

## Summary and Outlook

Amyotrophic lateral sclerosis is a progressive and fatal motor neuron degenerative disease with high socioeconomic significance, and it affects approximately 290,000–360,000 patients worldwide. Unfortunately, until recently, it is hard to diagnose ALS in the early stage, and treatment means are limited, and the treatment effect is unsatisfactory. How to resolve those difficulties which restrict the outcome of ALS patients is an urgent problem. EVs have great application potential in diagnosis, treatment, and gene delivery in ALS, and represent an innovative treatment strategy. Notably, on the one hand those vesicles can cross the BBB, on the other hand they can have similar effects of stem cells and additional delivery capacity for gene therapy. However, most of the gratifying research results come from basic experiments, thus no clinical improvement can be provided by existing research results. The research on the chemical constituents (e.g., protein, peptides, nucleic acids, and other small molecules) of EVs has developed slowly, and fewer studies reported the recommended doses of EVs in the treatment of ALS. Furthermore, the attempts that have been made using EVs for gene delivery to treat ALS are still limited. Meanwhile, we can not ignore that some limitations are still hampering the translation of EVs in clinical therapies, such as isolation and purification technologies, storage and stability, etc. (Ha et al., 2016). There is no unified standard for the above parameters so far. Biosafety, biocompatibility, biodegradability, and low immunogenicity are the fundamental requirements for brain drug delivery systems (Zheng et al., 2019). Since massive exogenous engineered EVs uptake by brain tissue with the specific distribution is not clear in humans or primates, that may cause potential risk, especially the EVs with additional membrane modifications by the introduction of foreign species (e.g., aptamers, antibodies, and peptides), may generate unwanted immunogenicity (Armstrong et al., 2017). Mammalian cell-derived EVs, plant-derived EVs, and exosome-mimics, either unmodified or engineered, can cross or bypass the BBB both *in vivo* and *in vitro*, but the BBB of mice and humans has some crucial species differences in the function, implicating that a series of basal and preclinical studies are needed to explore before clinical application (Pistono et al., 2021). Although there is a long way to go before the researchers, the current research on EVs brings new hope for patients with ALS, and should encourage further research efforts in this direction.

## Author Contributions

YJ and KW contributed to the conception and design of the study. YJ and YL wrote the first draft of the manuscript. CR, YW, and WH wrote sections of the manuscript. YJ proofread the manuscript. All the authors contributed to manuscript revision and approved the submitted version.

## Conflict of Interest

The authors declare that the research was conducted in the absence of any commercial or financial relationships that could be construed as a potential conflict of interest.

## Publisher’s Note

All claims expressed in this article are solely those of the authors and do not necessarily represent those of their affiliated organizations, or those of the publisher, the editors and the reviewers. Any product that may be evaluated in this article, or claim that may be made by its manufacturer, is not guaranteed or endorsed by the publisher.
